# Supplementary material for: Cytotoxicity of Human Hepatic Intrasinusoidal CD56bright Natural Killer Cells against Hepatocellular Carcinoma Cells
Source: Int J Mol Sci. 2019 Mar 28;20(7):1564. doi: 10.3390/ijms20071564 (PMC6480584; doi:10.3390/ijms20071564)

Supplementary Materials and Methods

Gene expression analysis

$CD3^-CD56^{bright}CD16^-$ and $CD3^-CD56^{dim}CD16^+$ cells from liver perfusate or peripheral blood from healthy volunteers were sorted using FACSAria II (BD Biosciences), and total RNA was isolated using TRIzol reagent (Invitrogen), following standard protocols. Transcriptomic analysis was performed using nCounter Human Immunology Panel v2 (NANOstring, Seattle, WA, USA) by PhileKorea, Co. (Daejeon, Korea). N=2-3 for each group.

Supplementary Table 1. Mean fluorescence intensity of the selected cell death receptors and activating ligands on HCC cell lines. Data are representatives of at least 3 independent experiments.

MFI	SNU398	Huh7
CD40	1603	2313
DR4	689	1986
DR5	1714	5318
FAS	21590	7571
MICA/B	2129	1171

Supplementary Table 2. Mean fluorescence intensity of the selected inhibitory/immune checkpoint ligands on HCC cell lines. Data are representatives of at least 3 independent experiments.

MFI	SNU398	Huh7
CD274	6465	15903
CD80	9265	23236
CD86	3526	9915
HLA-E	3450	8236
HLA-ABC	2102	4885

Supplementary Table 3. Expression of NK cell receptors on $CD56^{bright}$ and $CD56^{dim}$ PB NK n=3. Means±SEM are showed.

**%**	**$CD3^{-}CD56^{bright}CD16^{-}$**	**$CD3^{-}CD56^{dim}CD16^{+}$**	**Fold ($CD56^{dim}/CD56^{bright}$)**
NKG2D	98.46±1.24	94.33±1.28	0.95
CD253 (TRAIL)	60.46±2.59	21.5±0.79	0.35
CD178 (FASL)	62.23±4.82	56.33±2.08	0.9
CD279 (PD1)	7.57±2.55	12.81±2.93	1.69
CD152 (CTLA4)	0	1.01±0.08	

**MFI**	$CD3^{-}CD56^{bright}CD16^{-}$	$CD3^{-}CD56^{dim}CD16^{+}$	Fold ($CD56^{dim}/CD56^{bright}$)
NKG2D	38161.33±3467.71	19641.67±1106.89	0.51
CD253 (TRAIL)	6194±337.03	3541.33±242.6	0.57
CD178 (FASL)	5992±271.33	5574±52.16	0.93
CD279 (PD1)	1264±44.23	1522.66±32.21	1.2
CD152 (CTLA4)	1523±200.15	1790.33±75.18	1.17

Supplementary Table 4. Transcriptomic analysis of $CD56^{bright}$ $CD16^{-}$ and $CD56^{dim}$ $CD16^{+}$ HI NK cells.

▶ LP_CD16_minus vs. LP_CD16_plus

Gene Name	Accession #	Class Name	LP_CD16_minus vs. LP_CD16_plus
AHR	NM_001621.3	Endogenous	10.03
AICDA	NM_020661.1	Endogenous	-6.65
AIRE	NM_000383.2	Endogenous	-4.60
APP	NM_000484.3	Endogenous	2.08
ARG1	NM_000045.2	Endogenous	-5.40
ARG2	NM_001172.3	Endogenous	-3.99
ATM	NM_000051.3	Endogenous	-2.03
B3GAT1	NM_018644.3	Endogenous	-13.73
BATF	NM_006399.3	Endogenous	-2.95
BCL2	NM_000657.2	Endogenous	-3.26
BCL2L11	NM_138621.4	Endogenous	2.53
BLNK	NM_013314.2	Endogenous	3.09
BST1	NM_004334.2	Endogenous	3.82
BST2	NM_004335.2	Endogenous	3.14
BTLA	NM_181780.2	Endogenous	6.38
C1QA	NM_015991.2	Endogenous	-7.91
C1QB	NM_000491.3	Endogenous	-5.05
C1S	NM_001734.2	Endogenous	-9.31
C4A/B	NM_007293.2	Endogenous	-7.27
C6	NM_000065.2	Endogenous	-8.19
C8B	NM_000066.2	Endogenous	-5.46
C8G	NM_000606.2	Endogenous	-6.71
C9	NM_001737.3	Endogenous	-5.80
CAMP	NM_004345.3	Endogenous	-3.06
CARD9	NM_052813.4	Endogenous	-5.49
CASP10	NM_032977.3	Endogenous	-3.17
CASP8	NM_001228.4	Endogenous	-2.41
CCBP2	NM_001296.3	Endogenous	-3.76
CCL11	NM_002986.2	Endogenous	-6.52
CCL13	NM_005408.2	Endogenous	-14.35
CCL15	NM_032965.3	Endogenous	-3.57
CCL16	NM_004590.2	Endogenous	-4.68
CCL18	NM_002988.2	Endogenous	-9.50
CCL20	NM_004591.1	Endogenous	4.17
CCL22	NM_002990.3	Endogenous	-3.76
CCL23	NM_145898.1	Endogenous	-3.24

CCL24	NM_002991.2	Endogenous	-4.72
CCL26	NM_006072.4	Endogenous	-2.61
CCL7	NM_006273.2	Endogenous	-6.65
CCL8	NM_005623.2	Endogenous	-5.96
CCR10	NM_016602.2	Endogenous	-6.91
CCR5	NM_000579.1	Endogenous	6.01
CCR8	NM_005201.2	Endogenous	-3.76
CCRL1	NM_016557.2	Endogenous	-14.27
CD160	NM_007053.2	Endogenous	4.51
CD19	NM_001770.4	Endogenous	3.49
CD22	NM_001771.2	Endogenous	38.93
CD247	NM_198053.1	Endogenous	-2.05
CD27	NM_001242.4	Endogenous	9.22
CD274	NM_014143.3	Endogenous	-3.05
CD276	NM_001024736.1	Endogenous	-3.09
CD34	NM_001025109.1	Endogenous	-3.76
CD36	NM_001001548.2	Endogenous	2.49
CD3D	NM_000732.4	Endogenous	-2.92
CD3EAP	NM_012099.1	Endogenous	-2.38
CD4	NM_000616.4	Endogenous	2.99
CD40LG	NM_000074.2	Endogenous	2.93
CD45R0	NM_080921.3	Endogenous	6.39
CD55	NM_000574.3	Endogenous	-2.22
CD70	NM_001252.2	Endogenous	-3.76
CD74	NM_001025159.1	Endogenous	2.21
CD79A	NM_001783.3	Endogenous	8.21
CD80	NM_005191.3	Endogenous	-5.36
CD83	NM_004233.3	Endogenous	3.43
CD8A	NM_001768.5	Endogenous	-8.13
CD8B	NM_004931.3	Endogenous	-5.44
CD9	NM_001769.2	Endogenous	-2.93
CDH5	NM_001795.3	Endogenous	-3.76
CEACAM1	NM_001712.3	Endogenous	-2.10
CEACAM6	NM_002483.4	Endogenous	-2.86
CEACAM8	NM_001816.3	Endogenous	-8.45
CEBPB	NM_005194.2	Endogenous	-2.87
CFB	NM_001710.5	Endogenous	-2.17
CFP	NM_002621.2	Endogenous	2.04
CISH	NM_145071.2	Endogenous	-17.11
CLEC4E	NM_014358.2	Endogenous	4.29
CLU	NM_001831.2	Endogenous	-3.72
CMKLR1	NM_004072.1	Endogenous	-210.96

CSF1	NM_000757.4	Endogenous	-7.17
CSF1R	NM_005211.2	Endogenous	-3.24
CSF3R	NM_156038.2	Endogenous	2.06
CTLA4-TM	NM_005214.3	Endogenous	-14.90
CTLA4_all	NM_005214.3	Endogenous	-9.50
CTSG	NM_001911.2	Endogenous	-6.64
CUL9	NM_015089.2	Endogenous	-2.06
CX3CL1	NM_002996.3	Endogenous	-2.33
CX3CR1	NM_001337.3	Endogenous	-118.55
CXCL13	NM_006419.2	Endogenous	-3.99
CXCR1	NM_000634.2	Endogenous	-46.00
CXCR2	NM_001557.2	Endogenous	-41.65
CXCR6	NM_006564.1	Endogenous	12.40
DEFB1	NM_005218.3	Endogenous	-9.31
DEFB103A	NM_001081551.2	Endogenous	-6.43
DEFB103B	NM_018661.3	Endogenous	-7.05
DEFB4A	NM_004942.2	Endogenous	-14.43
EDNRB	NM_003991.2	Endogenous	-5.80
EGR2	NM_000399.3	Endogenous	-2.10
FADD	NM_003824.2	Endogenous	-5.92
FCAR	NM_133280.1	Endogenous	-8.35
FCER1A	NM_002001.2	Endogenous	3.16
FCGR2A/C	NM_201563.4	Endogenous	-2.36
FCGR3A/B	NM_000570.4	Endogenous	-403.97
FCGRT	NM_004107.4	Endogenous	2.93
FN1	NM_212482.1	Endogenous	-3.76
FOXP3	NM_014009.3	Endogenous	-2.30
GBP1	NM_002053.1	Endogenous	-5.09
GNLY	NM_006433.2	Endogenous	-15.94
GPR183	NM_004951.3	Endogenous	3.55
GZMB	NM_004131.3	Endogenous	-43.11
GZMK	NM_002104.2	Endogenous	10.37
HAMP	NM_021175.2	Endogenous	-3.99
HAVCR2	NM_032782.3	Endogenous	-6.28
HFE	NM_139011.2	Endogenous	-5.80
HLA-DMB	NM_002118.3	Endogenous	2.44
HLA-DQA1	NM_002122.3	Endogenous	-2.63
HLA-DQB1	NM_002123.3	Endogenous	-2.99
HLA-DRA	NM_019111.3	Endogenous	3.80
ICAM2	NM_000873.3	Endogenous	-2.02
ICAM4	NM_001039132.1	Endogenous	-3.54
ICAM5	NM_003259.3	Endogenous	-3.76

ICOS	NM_012092.2	Endogenous	-3.17
IDO1	NM_002164.3	Endogenous	4.27
IFIT2	NM_001547.4	Endogenous	-2.81
IFNA1/13	NM_024013.1	Endogenous	-5.49
IFNA2	NM_000605.3	Endogenous	-7.50
IFNB1	NM_002176.2	Endogenous	-7.20
IGF2R	NM_000876.1	Endogenous	-2.68
IKBKB	NM_001556.1	Endogenous	-2.97
IKBKE	NM_014002.2	Endogenous	-3.37
IKZF1	NM_006060.3	Endogenous	-2.13
IL10	NM_000572.2	Endogenous	-3.08
IL10RA	NM_001558.2	Endogenous	-2.12
IL12A	NM_000882.2	Endogenous	-2.73
IL12B	NM_002187.2	Endogenous	-3.12
IL12RB1	NM_005535.1	Endogenous	-4.01
IL13	NM_002188.2	Endogenous	-17.53
IL13RA1	NM_001560.2	Endogenous	4.91
IL17A	NM_002190.2	Endogenous	-4.25
IL17B	NM_014443.2	Endogenous	-4.09
IL17F	NM_052872.3	Endogenous	-12.21
IL19	NM_013371.3	Endogenous	-3.76
IL1B	NM_000576.2	Endogenous	3.07
IL1RAP	NM_002182.2	Endogenous	-2.16
IL1RL2	NM_003854.2	Endogenous	-8.77
IL1RN	NM_000577.3	Endogenous	3.24
IL2	NM_000586.2	Endogenous	-3.76
IL20	NM_018724.3	Endogenous	-11.97
IL21	NM_021803.2	Endogenous	-5.80
IL21R	NM_021798.2	Endogenous	-6.21
IL22	NM_020525.4	Endogenous	-8.17
IL22RA2	NM_181310.1	Endogenous	-3.17
IL23A	NM_016584.2	Endogenous	-4.71
IL23R	NM_144701.2	Endogenous	7.19
IL26	NM_018402.1	Endogenous	-2.93
IL27	NM_145659.3	Endogenous	-3.17
IL28A	NM_172138.1	Endogenous	-33.40
IL28A/B	NM_172139.2	Endogenous	-4.07
IL29	NM_172140.1	Endogenous	-4.99
IL2RA	NM_000417.1	Endogenous	5.60
IL2RB	NM_000878.2	Endogenous	2.36
IL3	NM_000588.3	Endogenous	-4.07
IL32	NM_001012633.1	Endogenous	-2.04

IL4	NM_000589.2	Endogenous	-10.22
IL5	NM_000879.2	Endogenous	-3.76
IL7	NM_000880.2	Endogenous	-2.33
IL8	NM_000584.2	Endogenous	2.52
IL9	NM_000590.1	Endogenous	-3.76
IRAK3	NM_007199.1	Endogenous	-2.18
IRF5	NM_002200.3	Endogenous	5.06
IRF8	NM_002163.2	Endogenous	4.60
ITGA2B	NM_000419.3	Endogenous	-6.12
ITGA5	NM_002205.2	Endogenous	-10.13
ITGAX	NM_000887.3	Endogenous	-2.97
ITLN2	NM_080878.2	Endogenous	-2.30
KCNJ2	NM_000891.2	Endogenous	-7.12
KIR3DL1	NM_013289.2	Endogenous	-16.02
KIR3DL2	NM_006737.2	Endogenous	-6.73
KIR3DL3	NM_153443.3	Endogenous	-6.91
KIR_Activating_Subgroup_1	NM_001083539.1	Endogenous	-24.81
KIR_Activating_Subgroup_2	NM_014512.1	Endogenous	-115.33
KIR_Inhibiting_Subgroup_1	NM_014218.2	Endogenous	-13.66
KIR_Inhibiting_Subgroup_2	NM_014511.3	Endogenous	-14.38
KLRAP1	NR_028045.1	Endogenous	-19.97
KLRC1	NM_002259.3	Endogenous	2.55
KLRC3	NM_007333.2	Endogenous	-2.20
KLRC4	NM_013431.2	Endogenous	-2.53
KLRG1	NM_005810.3	Endogenous	-12.14
KLRG2	NM_198508.2	Endogenous	-2.17
LAG3	NM_002286.5	Endogenous	-6.90
LAIR1	NM_002287.3	Endogenous	-2.61
LAMP3	NM_014398.3	Endogenous	-2.27
LCK	NM_005356.2	Endogenous	-2.54
LIF	NM_002309.3	Endogenous	3.01
LILRA1	NM_006863.1	Endogenous	-2.68
LILRA3	NM_006865.3	Endogenous	-3.76
LILRB1	NM_001081637.1	Endogenous	-8.00
LILRB2	NM_005874.1	Endogenous	-5.58
LILRB5	NM_001081442.1	Endogenous	-3.76
LITAF	NM_004862.3	Endogenous	-2.47
LTBR	NM_002342.1	Endogenous	4.22
LTF	NM_002343.2	Endogenous	-6.25
MAF	NM_005360.4	Endogenous	-6.37
MAP4K2	NM_004579.2	Endogenous	-2.38
MAPK11	NM_002751.5	Endogenous	-5.26

MARCO	NM_006770.3	Endogenous	-5.31
MASP1	NM_139125.3	Endogenous	-3.47
MASP2	NM_139208.1	Endogenous	-5.80
MBL2	NM_000242.2	Endogenous	-2.30
MME	NM_000902.2	Endogenous	-7.64
MR1	NM_001531.2	Endogenous	4.21
MRC1	NM_002438.2	Endogenous	-2.08
MS4A1	NM_152866.2	Endogenous	26.64
MSR1	NM_002445.3	Endogenous	-2.44
MUC1	NM_001018017.1	Endogenous	-3.76
NCF4	NM_000631.4	Endogenous	9.53
NFATC2	NM_012340.3	Endogenous	-2.49
NFATC3	NM_004555.2	Endogenous	-2.21
NFKBIA	NM_020529.1	Endogenous	2.31
NOS2	NM_000625.4	Endogenous	-6.37
NOTCH1	NM_017617.3	Endogenous	-2.05
NT5E	NM_002526.2	Endogenous	3.24
PAX5	NM_016734.1	Endogenous	5.38
PDCD1	NM_005018.1	Endogenous	-8.72
PDCD1LG2	NM_025239.3	Endogenous	-3.25
PDGFB	NM_033016.2	Endogenous	-4.52
PDGFRB	NM_002609.3	Endogenous	-136.33
PECAM1	NM_000442.3	Endogenous	2.06
PIGR	NM_002644.2	Endogenous	-15.88
PLA2G2A	NM_000300.2	Endogenous	-7.87
PLA2G2E	NM_014589.1	Endogenous	-23.29
PLAU	NM_002658.2	Endogenous	-3.17
POU2F2	NM_002698.2	Endogenous	-2.17
PPBP	NM_002704.2	Endogenous	-5.88
PRDM1	NM_001198.3	Endogenous	-2.41
PRF1	NM_005041.3	Endogenous	-4.60
PRKCD	NM_006254.3	Endogenous	2.29
PTAFR	NM_000952.3	Endogenous	-3.15
PTGS2	NM_000963.1	Endogenous	8.06
PTK2	NM_005607.3	Endogenous	6.23
RAG1	NM_000448.2	Endogenous	-4.86
RAG2	NM_000536.3	Endogenous	-2.17
RELB	NM_006509.2	Endogenous	6.66
RORC	NM_001001523.1	Endogenous	2.83
S100A8	NM_002964.3	Endogenous	3.69
S100A9	NM_002965.2	Endogenous	3.40
S1PR1	NM_001400.3	Endogenous	-6.16

SELE	NM_000450.2	Endogenous	-6.91
SELPLG	NM_003006.3	Endogenous	-19.67
SH2D1A	NM_001114937.2	Endogenous	2.00
SKI	NM_003036.2	Endogenous	-3.04
SLC2A1	NM_006516.2	Endogenous	-2.66
SOCS3	NM_003955.3	Endogenous	3.08
STAT3	NM_139276.2	Endogenous	-2.03
STAT5A	NM_003152.2	Endogenous	2.48
SYK	NM_003177.3	Endogenous	2.43
TAL1	NM_003189.2	Endogenous	-10.08
TBX21	NM_013351.1	Endogenous	-5.19
TCF7	NM_003202.2	Endogenous	12.31
TGFBI	NM_000358.2	Endogenous	3.46
THY1	NM_006288.2	Endogenous	-3.76
TIRAP	NM_148910.2	Endogenous	-2.93
TLR3	NM_003265.2	Endogenous	-10.39
TLR7	NM_016562.3	Endogenous	-4.86
TLR9	NM_017442.2	Endogenous	-4.18
TMEM173	NM_198282.1	Endogenous	-2.86
TNFAIP6	NM_007115.2	Endogenous	-2.41
TNFRSF11A	NM_003839.2	Endogenous	-2.67
TNFRSF13C	NM_052945.3	Endogenous	9.45
TNFRSF1B	NM_001066.2	Endogenous	-3.87
TNFRSF4	NM_003327.2	Endogenous	-5.80
TNFRSF8	NM_152942.2	Endogenous	-15.70
TNFRSF9	NM_001561.4	Endogenous	-6.79
TNFSF15	NM_001204344.1	Endogenous	-3.24
TNFSF4	NM_003326.2	Endogenous	-2.39
TNFSF8	NM_001244.3	Endogenous	3.96
VCAM1	NM_001078.3	Endogenous	-11.07
VTN	NM_000638.3	Endogenous	-13.22
XCL1	NM_002995.1	Endogenous	2.49
XCR1	NM_005283.2	Endogenous	-5.71
ZAP70	NM_001079.3	Endogenous	-2.24
GUSB	NM_000181.1	Housekeeping	2.48

Supplementary Figure 1. Cytotoxicity of PB NK cells against SNU398 and Huh7 cells. CD107a assay was performed with whole PBMC. Representative plots are displayed from 3 independent experiments.

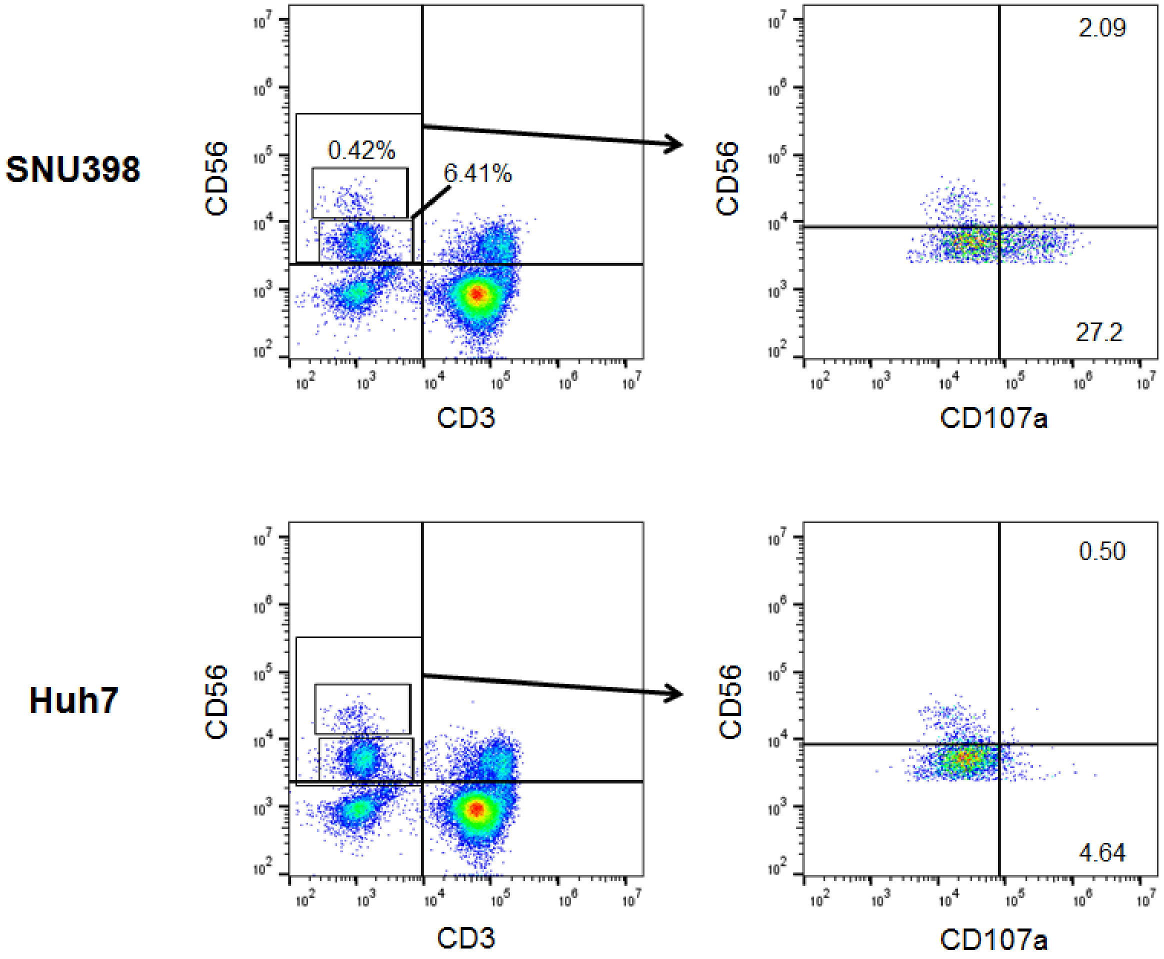

Supplementary Figure 2. Expression of NK cell receptors on CD56^{bright} and CD56^{dim} PB NK. Representative plots are displayed from 3 independent experiments.

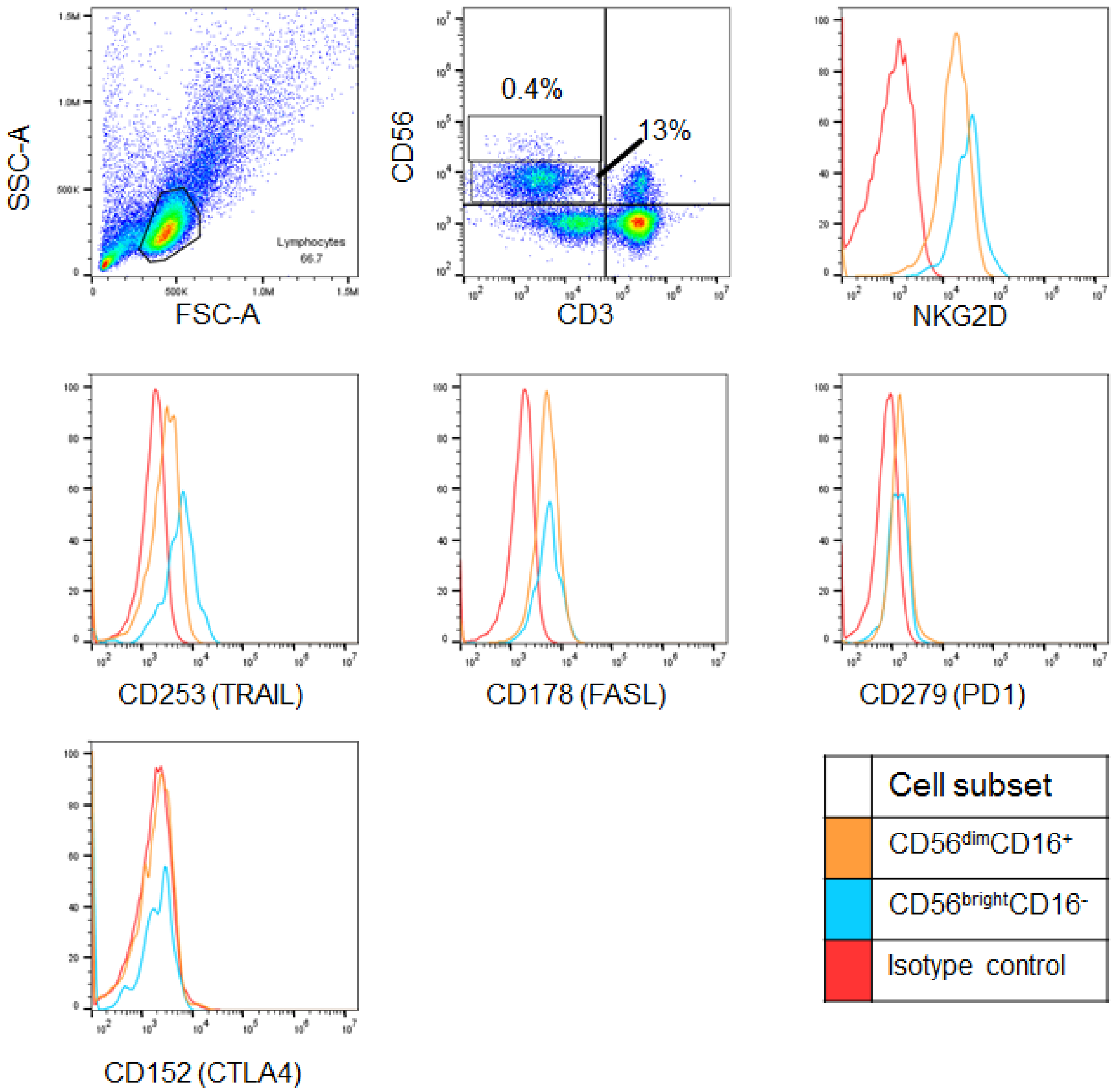

Supplement: Supplementary file 1 [file ijms-20-01564-s001.pdf]
